# Antimicrobial Resistance in Diverse *Escherichia coli* Pathotypes from Nigeria

**DOI:** 10.3390/antibiotics13100922

**Published:** 2024-09-26

**Authors:** Kenneth Nnamdi Anueyiagu, Chibuzor Gerald Agu, Uzal Umar, Bruno Silvester Lopes

**Affiliations:** 1 Department of Public Health Technology, Federal College of Animal Health and Production Technology, Vom 200273, Nigeria; anueyiagunk@gmail.com; 2 National Veterinary Research Institute, Vom 930103, Nigeria; chibuzoragu@nvri.gov.ng; 3 Department of Medical Microbiology and Parasitology, University of Jos, Jos 930105, Nigeria; uumar2@atbu.edu.ng; 4 School of Health and Life Sciences, Teesside University, Middlesbrough TS1 3BX, UK; 5 National Horizons Centre, Teesside University, Darlington DL1 1HG, UK

**Keywords:** *Escherichia coli*, *E. coli* O157:H7, Shiga-toxins, antimicrobial resistance, STEC, Diarrheagenic *E. coli*, *bla*_CTX-M_, ESβL

## Abstract

*Escherichia coli* is a gram-negative commensal bacterium living in human and animal intestines. Its pathogenic strains lead to high morbidity and mortality, which can adversely affect people by causing urinary tract infections, food poisoning, septic shock, or meningitis. Humans can contract *E. coli* by eating contaminated food—such as raw or undercooked raw milk, meat products, and fresh produce sold in open markets—as well as by coming into contact with contaminated settings like wastewater, municipal water, soil, and faeces. Some pathogenic strains identified in Nigeria, include Enterohemorrhagic (Verotoxigenic), Enterotoxigenic, Enteropathogenic, Enteroinvasive, and Enteroaggregative *E. coli*. This causes acute watery or bloody diarrhoea, stomach cramps, and vomiting. Apart from the virulence profile of *E. coli*, antibiotic resistance mechanisms such as the presence of *bla*_CTX-M_ found in humans, animals, and environmental isolates are of great importance and require surveillance and monitoring for emerging threats in resource-limited countries. This review is aimed at understanding the underlying mechanisms of evolution and antibiotic resistance in *E. coli* in Nigeria and highlights the use of improving One Health approaches to combat the problem of emerging infectious diseases.

## 1. Introduction

Theodor von Escherich, a German-Austrian paediatrician, was the first to isolate intestinal bacteria in neonates and infant faecal matter in 1857, which he termed ‘*Bacterium coli commune*’ [1]. This bacterium was originally called *Bacillus coli* in 1895 and renamed *Escherichia coli* in 1919 in honour of Escherich. The nomenclature was then formally acknowledged in 1958, forming *Escherichia* as a genus, with *E. coli* as its first species [2]. *E. coli* is a gram-negative, nonspore-forming, facultative anaerobic, rod-shaped bacteria belonging to the family Enterobacteriaceae, class Gammaproteobacteria, and phylum Pseudomonadota. These are normal intestinal commensals of humans and warm-blooded animals, and to date, ten species have been described, of which *E. adecarboxylata*, *E. albertii*, *E. blattae*, *E. coli*, *E. fergusonii*, *E. hermannii*, *E. marmotae*, *E. ruysiae*, *E. vulneris,* and *E. whittamii* are validly published under the International Code of Nomenclature of Prokaryotes (ICNP), whereas *E. faecium*, *E. hominis,* and *E. pseudocoscoroba* are not validly published [3].

*E. coli* is the most widely studied organism, a lactose fermenter with colonies on MacConkey agar, appearing bright pink with a typical diameter of 0.5–1 mm after overnight incubation at 37 °C/24 h [4]. The colony appearance on blood agar plates can vary from grey to white, transparent to opaque, and raised convex to flat. On Eosin Methylene Blue (EMB) agar, *E. coli* produces a characteristic green metallic sheen. This bacterium can survive in water for 4–12 weeks, and as a result, it is used as a faecal indicator organism for determining bacterial contamination in water because of the availability of simple, and affordable techniques [5]. Some pathotypes of *E. coli,* such as the *E. coli* O157:H7 strain, are sorbitol nonfermenters, which is a feature that can help it to be distinguished from other strains of *E. coli* [6].

Most *E. coli* strains are harmless and essential to the human digestive system. *E. coli* can synthesise some essential vitamins for human health, such as vitamin K and some B vitamins, and aids in the breakdown of food and the absorption of nutrients. Additionally, they guard the gut by preventing the colonisation of harmful bacteria through colonisation resistance and resource limitation [7].

Conversely, several *E. coli* strains are nonpathogenic, but pathogenic types can adversely affect people and cause food poisoning, urinary tract infections, septic shock, or meningitis. *E. coli* can be divided into two broad categories: commensal and pathogenic [8]. Commensal *E. coli* is crucial for maintaining normal intestinal bacteria, supports innate and adaptive immunity, and is also important for the gastrointestinal tract because it produces vitamin K and vitamin B12 [9]. Extraintestinal pathogenic *E. coli* (ExPEC) can be divided into many main genetic subgroups, such as A, B1, B2, C, D, E, and I. Group A often includes isolates from the colon, ileum, and duodenum [10]. Phylogroup B1 contains both commensal and some strains belonging to the Enterohemorrhagic *E. coli* (EHEC) pathotype. B2, C, D1, D2, and E represent intestinal pathogenic *E. coli* (InPEC), which relates mainly to diarrhoeal diseases. Many ExPEC belong to group B2 and are primarily associated with adult urinary tract infections (UPEC) and neonatal meningitis (NMEC) [11].

The main source of pathogenic *E. coli* variations’ capacity to cause intestinal or extraintestinal disease is their development of several specific virulence factors, including toxins, lipopolysaccharides, iron acquisition factors, adhesins, polysaccharide capsules, and invasins. These elements aid the organism’s ability to get past the host’s defences and infiltrate or colonise the organs.

*E. coli* is transmitted to humans primarily through the consumption of contaminated foods, such as raw or undercooked ground meat products and raw milk. An increasing number of outbreaks are associated with the consumption of fruits and vegetables (including sprouts, spinach, lettuce, coleslaw, and salad), whereby contamination may be due to contact with faeces from domestic or wild animals at some stage during cultivation or handling [12]. The reservoir of this pathogen includes cattle, sheep, goats, deer, pigs, horses, rabbits, dogs, cats, and birds, including chickens and turkeys [13]. Faecal contamination of water and other foods, as well as cross-contamination during food preparation (with beef and other meat products, contaminated surfaces, and kitchen utensils), can also lead to infection [14].

Apart from the virulence profile of *E. coli*, antibiotic resistance mechanisms of this bacterium are one of the most significant issues in recent research. Antibiotic resistance happens when bacteria evade the effect of antibiotics either by mutations in functional genes or by acquiring genes that can hydrolyse antibiotics and can also be transferred via plasmids between different strains [15]. One of the most clinically and epidemiologically significant mechanisms of antibiotic resistance in Enterobacteriaceae is the synthesis of carbapenemases, AmpC-type β-lactamases, and extended-spectrum β-lactamases (ESβLs) [16]. In this study, we review the prevalence of different *E. coli* pathotypes and their underlying mechanisms of antibiotic resistance in Nigeria.

### 1.1. Diarrheagenic E. coli Pathotypes (DEC)

According to the WHO Global Burden of Foodborne Diseases report, Diarrheagenic *E. coli* (DEC) causes approximately 200,000 deaths and over 300 million illnesses worldwide each year [17]. Approximately 40% of bouts of acute diarrhoea in children in underdeveloped countries are caused by diarrhoeagenic *E. coli* [18]. Additionally, they significantly contribute to both adult and paediatric diarrhoea in Nigeria [18]. Enterotoxigenic (ETEC), Enterohaemorrhagic (EHEC), Enteroinvasive (EIEC), Enteropathogenic (EPEC), Enteroaggregative (EAEC), Diffusely Adherent (DAEC), Cytolethal Distending toxin-producing (CDTPEC), and Cell-Detaching *E. coli* (CDEC) are the eight pathotypes of DEC strains that are currently known [18,19]. Each pathotype of DEC has a unique set of virulence factors encoded either on the plasmids or on the chromosomes. *E. coli* pathotypes cause substantial diarrhoeal illnesses in Nigeria. These infections are especially serious for children under five, as they increase morbidity and mortality and exacerbate public health issues in an environment where access to clean water and quality healthcare is frequently restricted. In a previous study from State Hospital Oke-Ogbo, Ile-Ife, Osun State, Nigeria, DEC was isolated from 90 (35.7%) of the 126 mother-child pairs, with 45 (35.7%) samples from children with diarrhoea and 45 (35.7%) from their mothers. The prevalence of DEC in children with diarrhoea was comparable to previous studies conducted in Ile-Ife in children with diarrhoea [20]. Shiga toxin-producing *E. coli* (STEC) was the most prevalent DEC subpathotype detected, accounting for 42 cases (16.7%). Of these, 32 STEC strains carried both the *stx1* and *stx2* genes. Other identified pathotypes included enterotoxigenic *E. coli* (ETEC) with 40 cases (15.9%), Enteroaggregative *E. coli* with 21 cases (8.3%), Enteropathogenic *E. coli* (EPEC) with 13 cases (5.2%), and Enteroinvasive *E. coli* (EIEC) in only 2 cases (0.8%), exclusively linked to isolates from mothers [20].

#### 1.1.1. Enterohaemorrhagic *E. coli* (EHEC)

One of the major foodborne infections caused by Enterohaemorrhagic *E. coli* O157:H7, frequently results in a high death rate among humans [21]. Common infections brought on by this bacterium have the potential to be fatal. Haemorrhagic colitis and other problems, including haemolytic uremic syndrome (HUS) and thrombotic thrombocytopenic purpura, are among the symptoms that susceptible people may experience [22,23]. Toxin tests and stool cultures are used for diagnosis. *E. coli* O157 was found to be one of the main causes of foodborne disability-adjusted life years, placing Nigeria in a subregion with the greatest prevalence of foodborne disease worldwide. According to Gambushe et al. [24], this infection is also common in South Africa and Ethiopia. Wells, water troughs, and bodies of water (such as ponds and streams) have all been shown to contain EHEC, which could be due to the contamination of water with ruminant faeces, which are common carriers of this pathogen. Nigeria is among the nations where 90 million people lack access to potable water, and 130,000 children under the age of five pass away every year from preventable waterborne illnesses as a result of government agencies’ disorganised efforts, lack of funding, and basic public education about hygienic practices, public health policies, and engaging with various stakeholders. The majority of people, especially those living in rural and suburban areas, use untreated water from wells and streams for domestic use, which puts them at risk of *E. coli* infection through the faecal-oral route [25,26]. Waterborne transmission, where 88.9% of *E. coli* isolates were of the EHEC pathotype, has been reported in Nigeria, both from contaminated drinking water and recreational waters [27].

Although it has also been found to survive for months in manure, beef and mutton are used in steaks; however, in Nigeria, meats are cooked thoroughly, so STEC outbreaks linked with undercooked meat are rare. Interpersonal contact is a crucial means of transmission via the faecal-oral pathway. There have been reports of an asymptomatic carrier condition in which a person can infect others yet exhibit no clinical symptoms of the illness. Another known risk factor for STEC infection is going to farms and other places where the general public may have direct contact with farm animals. Ruminants such as cattle, sheep, and goats are largely reared by pastoralists and agropastoralists, and common practice in the Fulani tribe can also be exposed to strains such as *E. coli* O157 [28].

Antibiotic therapy is generally not recommended for EHEC infections [29] because it is of no benefit [30], or even harm, in particular, but an increased risk of haemolytic uremic syndrome (HUS) development in patients treated with antibiotics during the initial period of diarrhoea [31]. Shiga toxin (Stx), the primary virulence component of EHEC implicated in the pathogenesis of HUS, is produced and/or released more frequently when antibiotics are used, which is one conceivable mechanism by which they raise the chance of developing HUS [32]. The goal of treating STEC is to remove the bacteria from the intestine without causing it to start producing toxins. Recently, STEC has been treated with different methods, such as antisera or monoclonal antibodies [33].

#### 1.1.2. Enterotoxigenic *E. coli* (ETEC)

ETEC is a primary enteric pathogen that is responsible for annual millions of diarrhoeal diseases, globally [34]. In the laboratory, ETEC is diagnosed when the microbe is cultured from faecal samples and tested for the presence of toxins *(LT*, *STh*, and *STp*) using phenotypic assays like dot blotting or through the use of conventional PCR [35]. Children less than 5 years old are vulnerable to ETEC, especially in developing countries like Nigeria, where the disease is endemic. ETEC accounted for approximately 100 million diarrhoea episodes and 60,000 mortalities in 2015 [34]. ETEC is also the major cause of travellers’ diarrhoea, affecting travellers visiting developing countries of the world [36]. In underdeveloped nations, where there is no infrastructure for the collection of human waste and the supply of clean drinking water, ETEC infections are caused by consuming contaminated food and water. Previous research has shown that ETEC may live in faeces for up to six months and that it often grows in water as biofilms, which gives it a better chance of surviving [37].

#### 1.1.3. Enteroinvasive *E. coli* (EIEC)

EIEC appears to be a less frequent cause of diarrhoeal sickness than other *E. coli* infections, however, this may be due to the methods employed to identify these organisms [38]. It is diagnosed in the laboratory by culturing stool samples and by the detection of EIEC pathogenicity genes by DNA hybridisation or PCR. EIEC produces a diarrhoeal illness that is indistinguishable from shigellosis. The majority of diarrhoeal illness cases that are caused by EIEC seem to be sporadic. *Shigella* spp. are closely linked to the EIEC, which uses Shigella-like genetic material to encode virulence components, including the type III secretion system (TTSS). The EIEC enters the intestinal cell, multiplies intracellularly, and extends into neighbouring intestinal cells via virulence proteins known as “invasins”. Cell death and occasionally bloody diarrhoea result from this process [38]. The majority of EIEC illness manifests clinically as watery diarrhoea and is difficult to recognise at the bedside from the many other causes of diarrhoea [38].

#### 1.1.4. Enteropathogenic *E. coli* (EPEC)

According to Lanata and Walter [39], Enteropathogenic *Escherichia coli* (EPEC) is a common strain of *E. coli* that causes persistent diarrhoea and is one of the main causes of paediatric *E. coli* infections worldwide [40]. Over the past few decades, the importance of EPEC as a pathogen has generally been neglected [41,42]. In contrast to more recent studies that relied on molecular techniques and/or adherence assays to identify EPEC, it is unclear whether the decrease in EPEC infections is the result of interventions, particularly breastfeeding promotion, or if earlier studies overestimated the relative contribution of these organisms in diagnosis based on O- or O: H-typing [43].

#### 1.1.5. Enteroaggregative *E. coli* (EAEC)

Enteroaggregative *E. coli* (EAEC) has gained increased attention in the last ten years as a potential cause of persistent, watery diarrhoea. According to Kaur et al. [44], EAEC is a rather diverse category of an emerging enteric pathogen connected to episodes of acute or chronic diarrhoea in children and adults worldwide. In both developing and wealthy nations, EAEC infection is a significant contributor to diarrhoea in both outbreak and nonoutbreak situations. Irritable bowel syndrome has recently been linked to EAEC, albeit this has not yet been proven [44].

#### 1.1.6. Diffusely Adherent *E. coli* (DAEC)

DAEC strains are a varied group of isolates that all exhibit diffuse adherence (DA) to epithelial cells in the conventional laboratory assay of adherence to HEp-2 or HeLa cells [45]. The production of adhesins encoded by a family of operons connected to afa/dra/daa is often the cause of the DA pattern of DAEC isolates.

#### 1.1.7. Cytolethal-Distending Toxin-Producing *E. coli* (CDTEC)

*Escherichia coli* strains obtained from patients with diarrhoea were shown to have the Cytolethal Distending toxin (CDT) in 1987 [46]. Since then, several pathogenic gram-negative bacteria have been found to express CDT, including *Aggregatibacter* (formerly *Actinobacillus*) *actinomycetemcomitans*, *Campylobacter* spp., *Escherichia* spp., *Haemophilus ducreyi*, *Helicobacter* spp., *Providencia alcalifaciens*, and *Shigella* sp. In in vitro studies, it has been shown to stop the proliferation of various cell lines [47]. Specific molecular techniques, like polymerase chain reaction (PCR), genotyping, and ELISA, are used to detect the presence of the genes encoding Cytolethal-Distending Toxin (typically *cdtA*, *cdtB*, and *cdtC* genes) [47].

#### 1.1.8. Cell-Detaching *E. coli* (CDEC)

When CDEC was initially identified, it was discovered to relate to diarrhoea in Australian aboriginal children [48]. Although no conclusive evidence links it to diarrhoea, it has also been discovered in Nigeria [49]. Notably, the majority of the CDEC isolates found in this investigation (87.0%) did not ferment lactose and were like a group of nonlactose-fermenting haemolytic strains that had been previously isolated from stool specimens in Somalia [50]. Lactose-negative CDEC strains may be significant pathogens in Africa [51]. Unfortunately, as is the case with most *E. coli* strains, their slow rate of lactose fermentation means that they are likely to be disregarded (or mistaken for Shigella or other nonlactose fermenters) in a lot of investigations. Additionally, diarrhoea may potentially be linked to Diffusely Adherent *E. coli* (DAEC). However, rates of infection vary by region in Africa [52].

The classification of pathogenic strains of *E. coli* is listed in Table 1, highlighting the symptoms, pathogenic mechanism, reservoirs, and epidemiological aspects for each of those groups. A characteristic feature of CDEC is its ability to cause the detachment of cells in tissue cultures. A cytotoxicity assay is performed where the *E. coli* isolate is applied to cultured epithelial cells in vitro.

## 2. Molecular Mechanisms of Antibiotic-Resistant *E. coli*

Globally, there is a growing issue with resistance to broad-spectrum β-lactams, which is caused by enzymes such as AmpC β-lactamases (AmpC), Metallo β-lactamases (MBL), and extended-spectrum β-lactamases (ESβL) [53]. The most common mechanism for β-lactam resistance in clinical isolates from the *Enterobacteriaceae* family is the synthesis of β-lactamases [54]. These groups of resistant *E. coli* have more diarrhoeagenic pathotypes than uropathogenic *E. coli* but could increase the risk for the latter [55]. A previous study on humans, cattle, and abattoir environments in Nigeria shows the high prevalence of ESβL-*E. coli* occurred in the abattoir environment (13.4%; n = 13), workers (41.2%; n = 40), and in livestock (45.4%; n = 44) [56].

### 2.1. Community- and Hospital-Acquired Antibiotic-Resistant E. coli

The rise of community-acquired antibiotic-resistant *E. coli* is primarily attributed to several factors. One key factor is the overuse and misuse of antibiotics in both human medicine and livestock production. It is noteworthy to mention that there are either no or little regulations for the use of antibiotics in Africa [57]. Widespread and irrational use of antibiotics creates an environment where bacteria can develop resistance mechanisms, rendering antibiotics ineffective at killing them. The implications of community-acquired antibiotic-resistant *E. coli* infections are significant. These infections are often more difficult to treat, as the bacteria are resistant to commonly used antibiotics, which can result in prolonged illness, increased healthcare costs, and, in some severe cases, life-threatening complications. Furthermore, treating these infections requires the use of more potent and costly antibiotics, which can further contribute to the emergence of antibiotic resistance. Globally, there is currently significant concern over the advent of strains of *E. coli* that produce extended-spectrum beta-lactamases (ESβLs) and community-acquired resistance to fluoroquinolones. A higher frequency of *E. coli* with elevated resistance to antibiotics is identified in patients suffering from complex urinary tract infections (UTIs) [58]. This is a result of the organisms acquired after being exposed to hospital settings, as well as the fact that many individuals with persistent abnormalities who suffer from recurring infections have taken numerous courses of antibiotics in the past [59]. In a previous study in Lagos, Nigeria, resistance to cephalosporins, including third- and fourth-generation types, ranged from 1.6% to 15% in community acquired *E. coli*. Specifically, resistance to cefpodoxime was found in 8.1% (20/247) of isolates, and resistance to cefepime was observed in 1.6% (4/247). Multidrug resistance (MDR) was identified in 69.6% (172/247) of the isolates. A subset of 48 *E. coli* resistant to at least five antimicrobials analysed by pulsed-field gel electrophoresis revealed seven distinct clusters, and multilocus sequence typing revealed 30 different sequence types, including ST131 [60].

Worldwide, UTIs are the most prevalent type of hospital-acquired illnesses. *E. coli* is the most frequent bacterium associated with UTIs. Different *E. coli* strains obtained from community and hospital sources may have different levels of antibiotic resistance and can often display multidrug-resistant phenotypes [61]. Studies show that ESβL-producing *E. coli* is becoming increasingly prevalent in hospitals and is of particular concern due to the emergence and spread of certain resistant clones [61,62]. In a tertiary hospital in Abuja, Nigeria, ESβL phenotype was found in 54 (50%) *E. coli* isolates, while *bla*_CTX-M-15_ genes occurred in 75 (70.1%) of the isolates [63].

### 2.2. Extraintestinal Pathogenic E. coli (ExPEC)

According to Nicolas-Chanoine et al. [64], ST131 is currently the most prevalent *E. coli* lineage discovered in extraintestinal pathogenic *E. coli* (ExPEC) isolates worldwide. Based on population genetics, ST131 is composed of several clades, including A, B, and C. Of these, clade C is the most widely distributed worldwide [65]. Compared to other traditional group B2 ExPEC isolates, almost all of the ST131 isolates are fluoroquinolone-resistant and are often reported to produce extended-spectrum β-lactamases, like CTX-M-15 [64]. Furthermore, ST131 *E. coli* isolates are thought to be extremely dangerous due to the range of infections they can cause in both community and hospital settings, as well as the quantity of virulence-associated genes they carry [64]. Multidrug-resistant ST131-*fim*H30, ST457-*fim*H145, ST405-*fim*H27, and ST95-*fim*H41 associated with *bla*_CTX-M-15_ were linked to the majority of ExPEC recovered from patients attending Abubakar Tafawa Balewa University Teaching Hospital (ATBUTH) in Nigeria [66].

### 2.3. Antibiotic-Resistant E. coli of Origins in Animals and the Environment

*E. coli* of animal origin exhibits resistance against a range of antibiotics due to overuse and abuse, including tetracyclines, aminoglycosides, β-lactams, and fluoroquinolones [67]. A recent report revealed that the extended-spectrum beta-lactamase *Escherichia coli* (ESβL-EC) strains from the human, chicken, and chicken market environments had the same sequence type (ST-155), indicating that there may have been a transmission between these hosts and the environment [68]. This finding also shows that cocolonization of antimicrobial-resistant *E. coli* from a shared source is a possibility. The ESβL-positive *E. coli* ST-155 represents a significant zoonotic strain that is responsible for the transmission to humans [69,70].

It is also important to note that ST-155 has been detected in *E. coli* isolates from water samples [69,70] and was detected in isolates from environmental samples [71]. Antibiotic-resistant *E. coli* concentrations in some wastewater treatment facilities and aquatic ecosystems reached 10^4^–10^5^ CFU/mL, which is more than what is needed for irrigation water usage, presenting a severe risk to public health [71].

## 3. Distribution of *E. coli* with Sources in Nigeria

### 3.1. Prevalence of Urinary Tract Infections Caused by E. coli

Among other studies, Olorunshola et al. [72] reported a 6% prevalence of *E. coli* from human stool samples in Nigeria (Table 2). This study, which was in a city of more than 13.4 million in 2000, was the least, while Mofolorunsho et al. [73] showed a higher prevalence of 70.3% from UTI in Anyigba town in Kogi State, which has less than 500,000 people in 2021.

### 3.2. E. coli in Food and Environment

Table 3 describes the prevalence of *E. coli* in food and environment. Besides human samples, *E. coli* can also be found in animals, animal products, plants, and environmental samples including water and soil. Adesiji et al. [81] showed a very high prevalence of the organism in fresh meat on sale, which could be due to faecal contamination. The prevalence of the organism in other animals and animal products has public health implications. Wastewater and dumpsites have a high prevalence, which is expected. Ruminant milk samples were seen to be contaminated by *E. coli* with prevalence rates of 44.8%, 43.1%, and 39.2% in cows, goats, and ewes, respectively [82,83]. Anueyiagu et al. [84] also isolated 22.73% of *E. coli* from domestic birds and 6.67% of *E. coli* from wild birds found in the same vicinity.

Fresh fruits and vegetables sold in Nigerian flea markets have been reported to be laden with micro-organisms such as *E. coli*. Maikai and Akubo [85] recorded a 21.3% prevalence, while Reuben and Makut [86] recorded a 17.5% prevalence.

Environmental sources of *E. coli* were reported by Edward et al. [87], Garba et al. [88], and Ijabani et al. [89], among many other authors.

**Table 3:** *E. coli* isolated from animal, plant, and environmental samples in Nigeria.

S/No	Source	Prevalence (n)	Author
1	Raw milk from cows	44.8% (640)	Anueyiagu et al. [82]
2	Raw milk from does	43.1% (206)	Anueyiagu et al. [83]
3	Raw milk from ewes	39.2% (206)	Anueyiagu et al. [83]
4	Fresh meat on sale	26% (300)	Adesiji et al. [81]
5	Fresh/Roasted beef	52.5% (300)/25.3% (150)	Dahiru et al. [90]
6	Poultry	31.8% (111)	Aworh et al. [68]
7	Wild birds	48.1% (160)	Oludairo et al. [91]
8	Vegetables	17.5% (40)	Reuben and Makut [86]
9	Fresh fruits	21.3% (108)	Maikai and Akubo [85]
10	Wastewater	62.4% (700)	Edward et al. [87]
11	Municipal water	45.5% (300)	Garba et al. [88]
12	Soil	20% (75)	Ijabani et al. [89]

## 4. Genotypes of *E. coli* in Nigeria and Other African Countries

The prevalence of Shiga toxigenic *E. coli* (STEC) differs by country. Most regions of the world, including several African nations, have reported cases of Shiga toxigenic *E. coli* infections [92]. One or more Shiga-toxin (Stx) strains are produced by human pathogenic STEC. These strains are classified into two groups: *stx1* (which includes the three gene variants *stx1a*, *stx1c*, and *stx1d*) and *stx2* (which includes seven unique variants stx2a, *stx2b*, *stx2c*, *stx2d*, *stx2e*, *stx2f*, and *stx2g*). Among these variations, stx2b and stx2e are connected to minor clinical symptoms or asymptomatic faecal carriage, while stx2a, stx2c, and stx2d are linked to severe disease [93,94,95]. The main STEC serogroups include *E. coli* O26, O45, O91, O103, O111, O113, O121, O128, O145, and O157, and they are all capable of producing Shiga toxins. In addition to *stx1* and *stx2*, certain isolates also have *eaeA* and/or *hlyA* [96]. The *hlyA* genes encode for cytolysin, while the *eae* gene encodes for intimin, which plays a vital role in the attaching and effacing (A/E) lesions on epithelial surfaces [97].

In 1990, in the South African city of Johannesburg, the first *E. coli* O157:H7 infection in humans was identified and recorded [24,98]. Nonetheless, in the Central African Republic in 1996, deadly pathogenic bacteria were discovered in patients suffering from hemorrhagic colitis. Following the emergence of bloody diarrhoea in Cameroon in 1998, STEC O157:H7 isolation in humans was reported [99]. There have been reports of pathogen isolation in Tanzania, Kenya, and Ethiopia in East Africa. In terms of the zoonotic disease epidemic on the African continent, Ethiopia is ranked second only to Nigeria [24]. According to the literature, pathogenic STEC that possess genes stx1, stx2, eaeA, and hylA are common in Nigeria. The *stx1*, *stx2*, *eaeA*, and *hylA* were isolated from beef and beef products, faeces, and meat of food-animal and beef abattoirs according to Fayemi et al. [100], Ojo et al. [101], and Ayoade et al. [102], respectively. The same *E. coli* variants were isolated from diarrhoeal patients from southwestern Nigeria [103]. In general, the evidence of STEC O157:H7 infection among the environment, animals, and humans, is obvious in Africa. In the laboratory, Sorbitol MacConkey agar is used to isolate STEC in Nigeria.

## 5. Antibiotic Resistance

The O157:H7 strain’s high resistance is recognised as one of the key elements that contributes to the possibility of the bacterium becoming more harmful. *E. coli* O157:H7 is found in food samples obtained from animals, particularly meat. It is extremely resistant to classes of widely used antibiotics, including quinolones, aminoglycosides, macrolides, cephalosporins, sulfonamides, fluoroquinolones, and tetracyclines. Antimicrobial resistance genes, such as those that confer resistance to beta-lactams (*bla*_CTX-M_), broad-spectrum penicillins/beta-lactams (*bla*_SHV_), gentamicin (*aac(3)-IV*), sulphonamide (*sul1*), tetracycline (*tet*A and *tet*B), and trimethoprim (*dfrA1*), are among those that enable resistance. However, it has been shown that the antimicrobial resistance of STEC strains is caused by aminoglycoside resistance genes (aadA1) [104]. Investigating the distribution and prevalence of antimicrobial-resistance variables and antimicrobial resistance patterns of *E. coli* O157:H7 strains acquired from raw beef, goat, chicken, and turkey meats is crucial given the ambiguity surrounding the dissemination of *E. coli* O157:H7 in raw meat samples [105].

Numerous studies conducted across the African continent have identified and documented resistance of *E. coli* O157:H7 to a range of antimicrobial drugs. For instance, multidrug-resistant *E. coli* O157:H7 was isolated from humans, animals, and the environment in Egypt, and its detection and documentation have been reported [99]. A multidrug-resistant *E. coli* O157:H7 was found and claimed to have been isolated from a cow in South Africa. In Nigeria, similar outcomes have been documented. Antimicrobial drugs’ severe overuse in treating *E. coli* O157:H7 infections is one of the main causes of concern [99]. This leads to multidrug resistance, which is not only concerning because it does not seem to be given much attention, but it also aids in the selection of resistance genes [99].

Given the advent of CTX-M-type extended-spectrum β-lactamases (ESβLs), recent reports in the literature indicated that *E. coli* is emerging as the Enterobacteriaceae species most impacted by ESβLs. It has been discovered that *bla*_CTX-M_ bacteria can have virulence genes in addition to resistance genes. Out of nine UTI samples collected from patients in Southwest Nigeria, six were positive for *bla*_CTX-M_ [103], and in Minna Metropolis of Nigeria, 60% of 10% of *E. coli* isolated from urine harboured *bla*_CTX-M_ [106]. Of the 175 ESβL-*E. coli* isolates from an orthopaedic hospital in Lagos, 48 (27.4%) were resistant to carbapenems (15 *E. coli* and 33 *K. pneumoniae*) [107]. In ruminant mastitis, the genetic characterisation revealed a higher prevalence of *bla*_CTX-M_ (24.39%) than *bla*_TEM_ (12.19%) in the bovine milk samples analysed [84]. A total of 440 samples obtained from Plateau and Kano states, consisting of 238 samples of fresh produce, 84 samples of irrigation water, and 118 samples of soil/manure, 32/440 samples were found to contain *E. coli* O157, with Kano State having the highest detection rate at 18/230 (12.2%). Thirty-two out of the thirty-two (93.8%) confirmed isolates had at least one antibiotic resistance, spread across seven distinct multidrug resistance pathways [108].

Additionally, it has been noted that the majority of *bla*_CTX-M_ from *E. coli* strains implicated in outbreaks across various nations also demonstrated the presence of additional antibiotic resistance genes, including *bla*_OXA-1_, *bla*_TEM-1_, *tet*A, *aac(6)-Ib*, and *aac(3)-II*, as well as occasionally a class 1 integron [109].

## 6. Conclusions

Nigeria and Africa face serious public health issues because of the spread of *E. coli-resistant* strains. Due to a lack of access to good healthcare, overuse and misuse of antibiotics, and use of antibiotics without a prescription, AMR can spread quickly throughout Nigerian communities. In addition to its enhanced virulence, highly pathogenic and antibiotic-resistant *E. coli* poses a severe hazard to food systems. These infections have been linked to one or more genetic factors of pathogenicity and pose a risk to human health. As a result of this research, policymakers should create a more proactive strategy to reduce the rise in *E. coli* infections in Nigeria. Therefore, to reduce the spread of potentially harmful or resistant *E. coli* strains, surveillance, low-cost diagnosis, and stringent hygiene practices of food (of animal and plant origin) should be continuously monitored. In general, individuals should prepare and consume food with greater hygiene standards. This assessment encourages Nigeria and other African nations to write and adopt new legislative measures for infectious diseases.

## Figures and Tables

**Table 1:** Classification and features of different *E. coli* pathotypes.

Pathotypes	Symptoms	Pathogenic Mechanism	Virulence Factors	Reservoirs	Epidemiological Aspects
Enterohaemorrhagic (EHEC)	Mild to severe diarrhoea frequently containing blood, haemorrhagic colitis, (haemolytic uremic syndrome)	Attaching effacing and production of potent verocytotoxins (Stx) that target renal structures (kidney failure).	Shiga toxin, type III secretion system (T3SS), intimin, Esp proteins, hemolysin	Zoonotic (ruminants, water)	In developed countries, most infections are in children or the elderly. Treated by intravenous fluids, corticosteroids, plasma filtration/exchange, dialysis (no antibiotics).
Enterotoxigenic (ETEC)	Mild to severe watery self-limiting diarrhoea	Possess either or both heat liable (LT) and heat-stable toxins (ST) like those produced by *Vibrio cholera*	Heat-labile toxin(LT), Heat-stable toxin (ST), Colonization factors (CFs)	Zoonotic (contaminated food and water)	Associated with childhood/traveller’s diarrhoea in developing countries treated by oral rehydration solution.
Enteroinvasive (EIEC)	Watery to dysentery-like diarrhoea—with blood and mucus in faeces, fever, abdominal cramps, inflammatory colitis	Ability to invade and replicate in intestinal epithelial cells related to *Shigella* spp.	Invasion plasmid antigens (Ipa proteins), Plasmid-encoded type III secretion system, actin polymerisation	Poor sanitary practices, food/waterborne	More important in developing countries with high morbidity and mortality of children.
Enteropathogenic (EPEC)	Watery diarrhoea, vomiting	Attaching-effacing lesions. Localised clusters adhere to the surface of cells.	Bundle-forming pili (BFP), type III secretion system (T3SS), intimin and Tir	Zoonotic (water, ruminants, chicken, faeces)	Associated with infantile diarrhoea (nurseries), especially in developing countries.
Enteroaggregative (EAEC)	Acute watery diarrhoea sometimes containing blood and mucous, mild fever, abdominal cramps, nausea, vomiting	Adherence by an aggregative adherence fimbria (AAF). Release of enterotoxin homologous to *Shigella flexneri*	Aggregative adherence fimbriae (AAF), enteroaggregative heat-stable toxin (EAST1), plasmid-encoded toxin (Pet), flagellin	Largely unknown, new-class foodborne outbreaks	Implicated with persistent diarrhoea (>14days) in developing and developed countries.
Diffusely adherent (DAEC)	Acute diarrhoea, vomiting, and sometimes fever	Fimbrial uniform adhesion-host cell elongates and wraps around the adherent bacteria	Fimbriae (F1845 and Afa/Dr adhesins), disruption of tight junctions, induction of host cell elongation	Largely unknown –new class	DAEC associated with infantile diarrhoea.
Cytolethal-distending toxin-producing *E*. *coli* (CDTEC)	Watery diarrhea; could progress to bloody diarrhoea, abdominal pain, and fever.	Produces a toxin called cytolethal distending toxin (CDT) which has DNase activity, meaning it can damage DNA within the host cells.	Cytolethal distending toxin (Cdt), cell death induction	Humans and animals (livestock and wild)	Affects individuals of all ages, but young children, the elderly, and immunocompromised individuals are at higher risk of developing severe disease.
Cell-Detaching *E. coli* (CDEC)	Watery diarrhoea, which can sometimes progress to more severe forms such as bloody diarrhoea. Abdominal pain, nausea, and vomiting may also be present.	CDEC strains produce a toxin or factors that cause the detachment of epithelial cells from the intestinal lining.	Cell-detaching toxin (Cdt), disruption of the actin cytoskeleton	The primary reservoir for CDEC is believed to be humans, although it may also be present in animals.	CDEC can affect individuals across all age groups, but children, especially those under five years old, are more susceptible to infections. The elderly and immunocompromised individuals are also at higher risk.

**Table 2:** *E. coli* isolated from humans in Nigeria.

S/No	Source	Prevalence (n)	Author
1	Human stool sample (DEC)	6% (100)	Olorunshola et al. [72]
2	Hospital-acquired (UTI and surgical site infections)	19.55% (22,941)	Ige et al. [74]
3	UTI in Abuja	37% (6763)	Iregbu and Nwajiobi [75]
4	UTI in Southwest	39.69% (514)	Oladeinde et al. [76]
5	UTI in Southeast	18.8% (266)	Okafor and Nweze [77]
6	UTI in North-Central	70.3% (200)	Mofolorunsho et al. [73]
7	UTI in Northwest	68.7% (128)	Muhammed et al. [78]
8	UTI in Northeast	41% (1590)	Ohieku and Magaji [79]
9	UTI in South-South	40% (300)	Ojezele [80]

## Data Availability

No new data were created or analysed in this study.
